# The effect of epigenetic aging on neurodegenerative diseases: a Mendelian randomization study

**DOI:** 10.3389/fendo.2024.1372518

**Published:** 2024-05-10

**Authors:** Jingqi Fan, Qing Liu, Xin Liu, Mengjiao Gong, Ian I. Leong, YauKeung Tsang, Xiaoyan Xu, Suying Lei, Lining Duan, Yifan Zhang, Muxi Liao, Lixing Zhuang

**Affiliations:** ^1^ Institute of Neurology, Guangzhou University of Chinese Medicine, Guangzhou, China; ^2^ The First Affiliated Hospital of Chinese Medicine, Guangzhou University of Chinese Medicine, Guangzhou, China

**Keywords:** epigenetic aging, neurodegenerative diseases, Mendelian randomization study, Alzheimer’s disease, Parkinson’s disease, Multiple Sclerosis

## Abstract

**Background:**

Aging has always been considered as a risk factor for neurodegenerative diseases, but there are individual differences and its mechanism is not yet clear. Epigenetics may unveil the relationship between aging and neurodegenerative diseases.

**Methods:**

Our study employed a bidirectional two-sample Mendelian randomization (MR) design to assess the potential causal association between epigenetic aging and neurodegenerative diseases. We utilized publicly available summary datasets from several genome-wide association studies (GWAS). Our investigation focused on multiple measures of epigenetic age as potential exposures and outcomes, while the occurrence of neurodegenerative diseases served as potential exposures and outcomes. Sensitivity analyses confirmed the accuracy of the results.

**Results:**

The results show a significant decrease in risk of Parkinson’s disease with GrimAge (OR = 0.8862, 95% CI 0.7914–0.9924, *p* = 0.03638). Additionally, we identified that HannumAge was linked to an increased risk of Multiple Sclerosis (OR = 1.0707, 95% CI 1.0056–1.1401, *p* = 0.03295). Furthermore, we also found that estimated plasminogen activator inhibitor-1(PAI-1) levels demonstrated an increased risk for Alzheimer’s disease (OR = 1.0001, 95% CI 1.0000–1.0002, *p* = 0.04425). Beyond that, we did not observe any causal associations between epigenetic age and neurodegenerative diseases risk.

**Conclusion:**

The findings firstly provide evidence for causal association of epigenetic aging and neurodegenerative diseases. Exploring neurodegenerative diseases from an epigenetic perspective may contribute to diagnosis, prognosis, and treatment of neurodegenerative diseases.

## 1 Introduction

Neurodegenerative diseases are a class of multifactorial diseases characterized by progressive loss of neuronal structure and function (1). Neurodegenerative diseases can affect cognitive function, mental state and motor function of patients, which lead to constant loss of essence of being. World Health Organization (WHO) reports that neurodegenerative diseases will overtake cancer and become the second leading cause of death by 2040 (2). Although there is no unified standard for the classification of neurodegenerative diseases, Alzheimer’s disease (AD), Parkinson’s disease (PD), and Multiple Sclerosis (MS) are predominantly common neurodegenerative diseases (3).

However, effective treatment and prevention measures of neurodegenerative disorders confront considerable hurdles due to unclear pathogenesis. Among heterogenous risk factors for neurodegeneration diseases, aging has by far the greatest impact (3). Of interest, although the rate of chronological aging is constant across individuals, there are individual variances in risk of neurodegenerative diseases. Even in aging adults with neurodegenerative disorders, disease progression and clinical phenotype still varies. Variability was also observed among family members who possessed identical pathogenic characteristic for neurodegenerative diseases, even among individuals with a strong hereditary background (4). Aging may involve more than a change in chronological age.

The measurement of biological age can be conducted at the epigenetic molecular level, particularly through utilization of DNA methylation (DNAm) age (5). The epigenetic clock was established by integration of DNAm values derived from a wide range of cytosine-phosphate-guanine base pairs (CpGs), which were chosen by supervised machine learning methodologies. Horvath developed the first epigenetic clock based on DNAm, which exhibited robust associations with chronological age. Hannum established a blood-based DNAm age algorithm consisting of 71 CpGs, which effectively recorded alterations in chronological age (6). Subsequently, many other DNA methylation-based ‘clocks’ have been developed. A variety of studies have identified associations between epigenetic aging and mortality, and other characteristics associated to health (7–9).

Some studies found that there is association with disease progression and clinical outcome of neurodegenerative diseases (10–16). Compared with age-matched controls, cortical tissue from patients with AD show modest DNA methylation changes in genes related in beta-amyloid proteins. A systematic review has shown that several epigenetic clocks were used to evaluate progression and pathology of AD and PD (17). Primarily, the Horvath clock was used for evaluating disease progression (18). A recent research of gene co-expression networks in patients with AD revealed the presence of epigenetic clocks-related genes, with enrichment of 50 distinct pathways (15). In general, there is a correlation between rapid DNAm age and a younger age of onset as well as quicker progression in several diseases, including AD (17).

Epigenetic aging may explain heterogeneity and homogeneity of various types of neurodegenerative diseases that will eventually dominate the aging brain. However, it remains unclear how epigenetic aging influences such an association. Thus, developing specific mechanisms between epigenetic aging and neurodegenerative disease is conducive to develop patient-tailored preventions and interventions. Furthermore, it may be useful in differentiating and diagnosing various forms of neurodegenerative disorders.

According to the latest study about polygenic risk for biomarkers of aging, the second-generation epigenetic clocks, GrimAge and PhenoAge, DNAm plasminogen activator inhibitor-1 (PAI1) levels and granulocyte proportion have a strong association with aging (19). A study has also found epigenetic factors are associated with the aging-related cognitive decline (20). They found that cognitive dysfunction had association with older Pheno Age progression and a faster Dunedin PACE (20). Therefore, we propose a hypothesis that these epigenetic genes may reveal specific mechanisms between aging and various kinds of neurodegenerative diseases.

As for causality between exposures and outcomes in diseases, mendelian randomization employs genetic variants as bridge variables to determine whether a risk factor causally influences the occurrence of diseases (21). Research of MR in neurodegenerative diseases is gradually receiving attention. Further studies on these heterogeneities may give insights to a specific mechanism between aging and neurodegenerative diseases, perhaps leading to early prevention, timely diagnosis, and efficient therapy.

Therefore, we conducted an MR study to examine causal effects between epigenetic aging and neurodegenerative diseases, such as AD, MS and PD.

## 2 Methods

### 2.1 Study design

**Figure 1:**
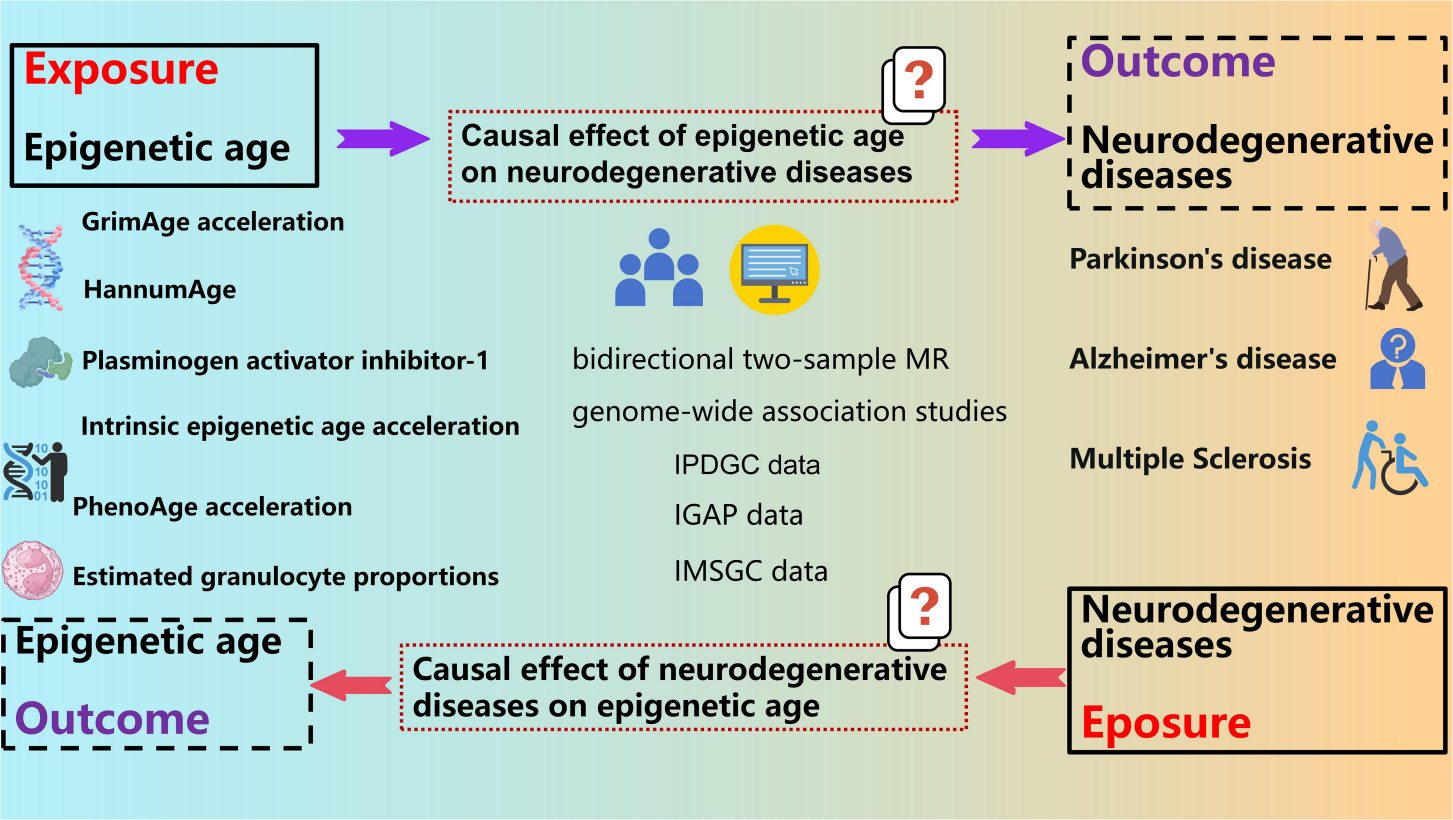
Study design of causal association of epigenetic aging and neurodegenerative diseases.

Our study employed a bidirectional two-sample Mendelian randomization (MR) design to assess the potential impact of epigenetic age on neurodegenerative diseases susceptibility. To acquire primary data, we utilized publicly available summary datasets from several genome-wide association studies (GWAS). Our investigation focused on multiple measures of epigenetic age as potential exposures, while the occurrence of neurodegenerative diseases served as the outcome of interest. In order to establish instrumental variables (IVs), we carefully selected specific single-nucleotide polymorphisms (SNPs) that exhibited strong associations with various aspects of epigenetic age. By ensuring adherence to the three key assumptions of the MR framework - (i) the genetic instrumental variables are correlated with the exposure (epigenetic age), (ii) the genetic instrumental variables are independent of potential confounders, and (iii) the genetic instrumental variables solely impact the outcome (neurodegenerative diseases) through the exposure (epigenetic age) - our objective was to examine the bidirectional causal association between epigenetic age and the risk of neurodegenerative diseases (**Figure 1**).

### 2.2 Summary data resources

#### 2.2.3 Epigenetic age

The study encompassed a diverse range of participants, with 57.3% being female, and the findings provide valuable insights into the methods employed for analyzing biological aging through genome-wide association studies. Age-adjusted DNA methylation-based estimates of HannumAge, Intrinsic Horvath age, PhenoAge, GrimAge, plasminogen activator inhibitor-1 levels were calculated using the Horvath epigenetic age calculator software https://www.ebi.ac.uk/gwas/publications/34187551 (19).

#### 2.2.4 Neurodegenerative diseases

For neurodegenerative diseases, a recent PD GWAS meta-analysis from the International Parkinson’s Disease Genomics Consortium (IPDGC) including 3 previously reported GWAS studies, 13 new datasets, as well as UKB proxy-case data (excluding 23and Me) was used as the PD source (33,674 cases, and 449,056 controls).

For AD, we used the recently published GWAS data, which is an International Genomics of Alzheimer’s Project (IGAP) meta-analysis of stage 1 as AD data in which 63,926 individuals were included (21,982 cases and 41,944 controls) from four consortiums as primary data for analysis. In addition, summary-level data for MS was retrieved from the shared data set by the most recent publication of the International Multiple Sclerosis Genetics Consortium(IMSGC) which included 47,429 MS cases and 68,374 controls. In this GWAS, 233 statistically independent genome-wide significant SNPs were found to be associated with MS susceptibility, explaining about 39% of the genetic predisposition to MS.

### 2.3 Selection of instrumental variables

**Figure 2:**
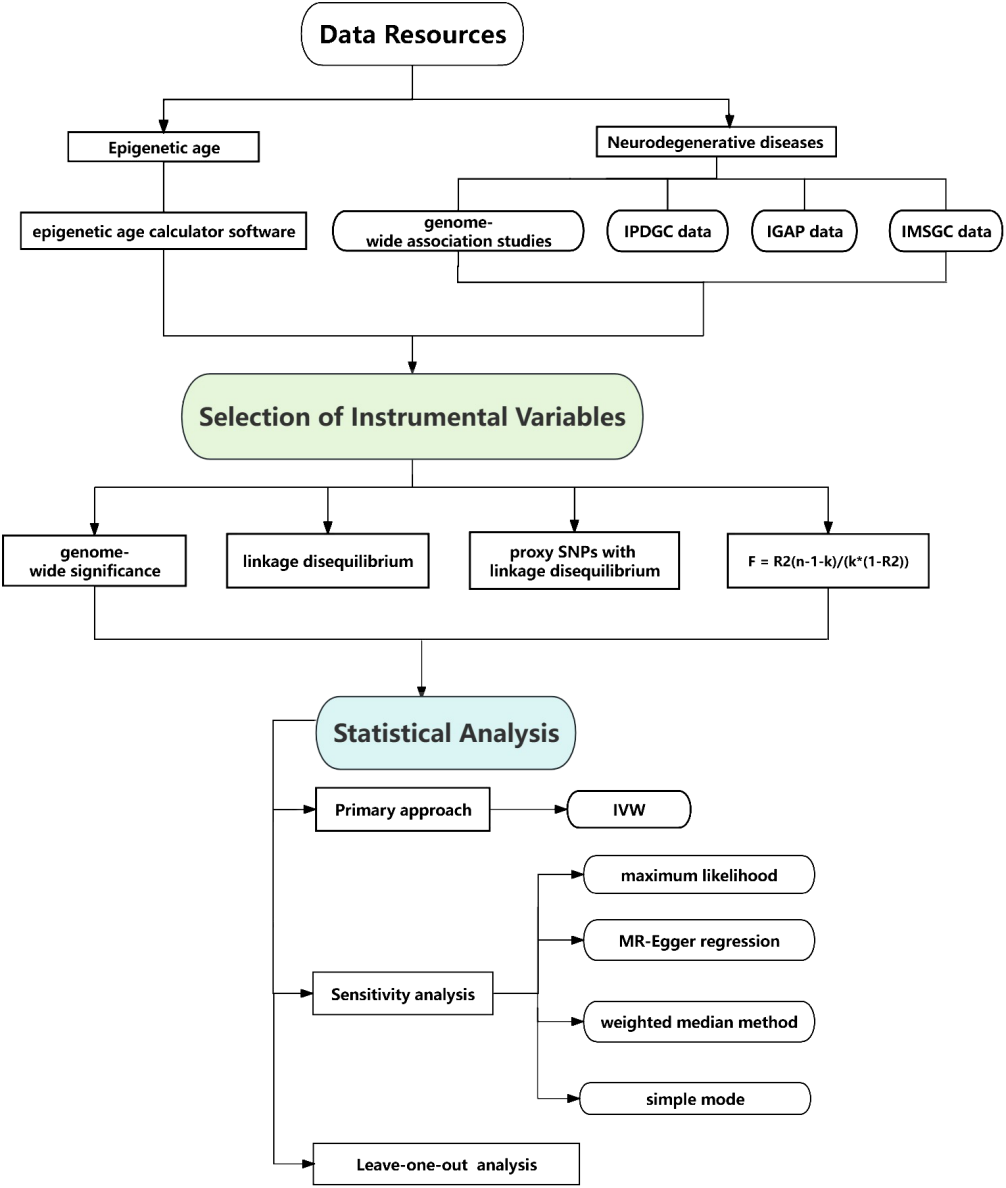
Flow chart of causal association of epigenetic aging and neurodegenerative diseases.

In order to identify genetic predictors associated with epigenetic age characteristics, we implemented a stringent quality control procedure. We applied a strict threshold of genome-wide significance (*P* < 5 × 10 ^-8^) to identify highly significant SNPs that are associated with both epigenetic age and neurodegenerative diseases. To ensure compliance with the assumptions of MR, we conducted a linkage disequilibrium (LD) analysis using data from the 1,000 Genomes Project, focusing on individuals of European ancestry. SNPs that did not meet the criteria (R^2^ < 0.001, clumping distance = 10,000 kb) were excluded from further analysis. Furthermore, we removed palindromic SNPs due to uncertainties regarding their alignment in the same direction for both the exposure and outcome variables in the neurodegenerative diseases genome-wide association studies. Additionally, SNPs with a minor allele frequency (MAF) below 0.01 were excluded from the analysis. In cases where SNPs associated with the exposure variable were missing in the outcome GWAS dataset, we selected proxy SNPs with a high level of linkage disequilibrium (r^2^ > 0.80) to ensure comprehensive coverage. To assess the strength of the instrumental variables, we calculated the F statistic using the formula F = R^2^(n-1-k)/(k*(1-R^2^)), where K is the number of instrumental variables, n is the sample size, R^2^ represents the proportion of variance explained by the instrumental variables, and n represents the sample size. An *F* statistic value below 10 indicates a higher likelihood of weak instrument bias, which highlights the need for cautious interpretation of the findings (**Figure 2**).

### 2.4 Statistical analysis

We employed inverse variance weighted (IVW) method as the primary approach for analyzing the MR data. In order to ensure the robustness of our findings, we also conducted several sensitivity analyses using alternative methodologies. These included maximum likelihood, MR-Egger regression, weighted median method, simple mode, and weighted mode method. Although some of these methods did not yield statistically significant results, we considered the findings positive if the IVW method produced significant results (*p* < 0.05) and the direction of the beta values remained consistent. To assess the impact on neurodegenerative diseases, we calculated odds ratios (OR) along with 95% confidence intervals (CIs), using a significance threshold of *p* < 0.05. Heterogeneity was evaluated using Cochran’s Q test for the IVW and MR-Egger estimates. To investigate potential pleiotropic bias, we utilized the MR-Egger regression technique. Furthermore, we performed a systematic “leave-one-out” analysis to evaluate the stability of our results, sequentially excluding each single-nucleotide polymorphism (SNP) to assess its influence on the overall findings. Additionally, a reverse MR analysis was performed to examine potential causal effects of neurodegenerative diseases on epigenetic age, following the same protocol as the two-sample MR. All statistical analyses were conducted using the Two Sample MR package (version 0.5.5) in the *R* software environment (version 4.0.3). These rigorous analytical approaches were employed to ensure the reliability and validity of the study’s outcomes (**Figure 2**).

## 3 Results

### 3.1 Selection of genetic instruments

To investigate the association between epigenetic age and the risk of neurodegenerative diseases, we conducted an MR analysis involving six specific epigenetic age traits. We ensured the use of robust genetic instruments (*p*< 5 × 10^-8^) to establish the independence of these traits (r^2^ < 0.01) by excluding palindromic single nucleotide polymorphisms (SNPs). The instrumental variables exhibited F-statistics that were all significantly greater than 10, indicating the absence of weak instrument bias. These measures were implemented to ensure the reliability and validity of our findings (**Supplementary Table 3**).

### 3.2 Causal effect of epigenetic age on neurodegenerative diseases

**Figure 3:**
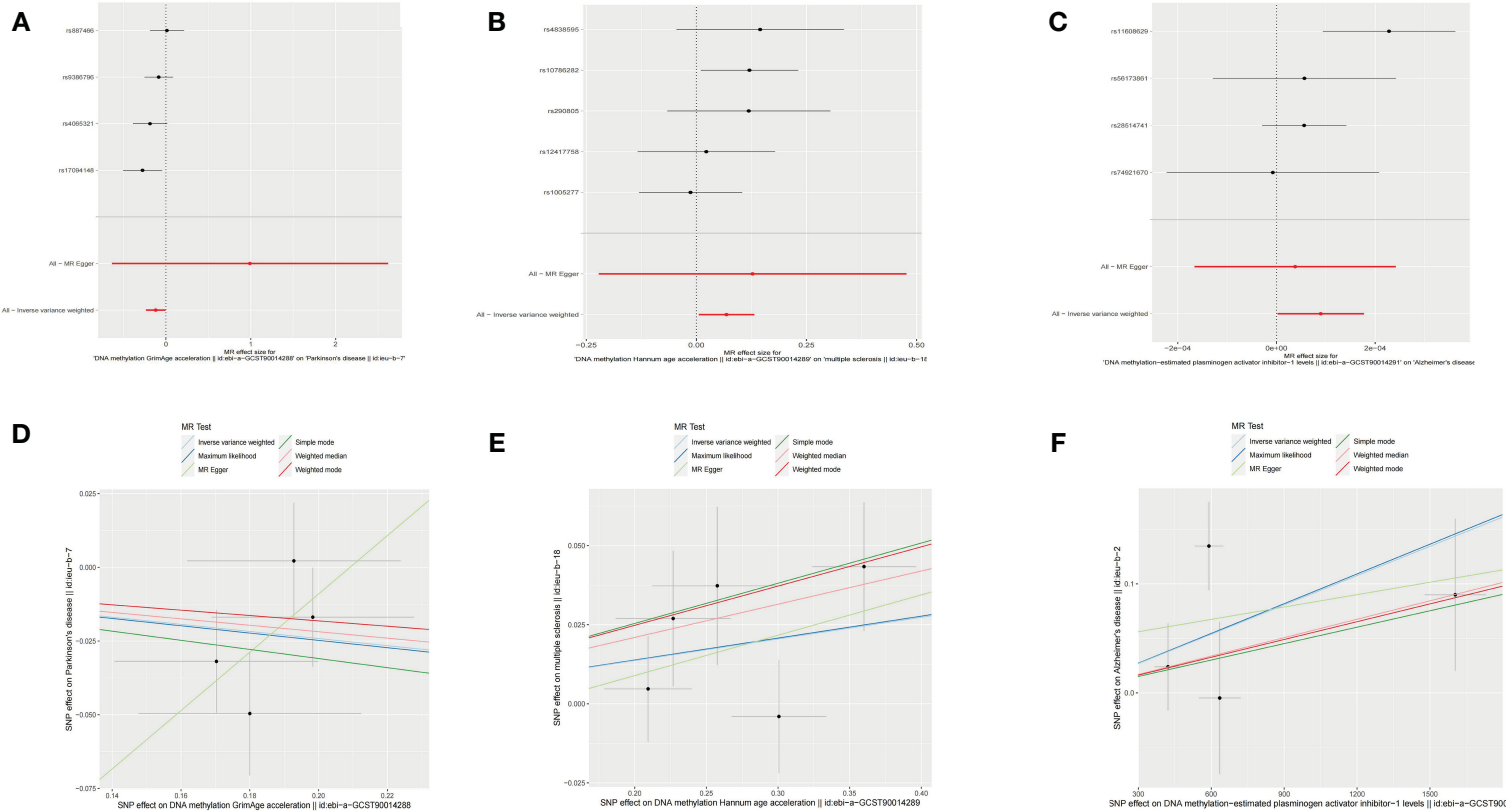
Causal effect of epigenetic age on neurodegenerative diseases. **(A)** Forest figure of DNA methylation GrimAge for PD risk. **(B)** Forest figure of DNA methylation HannumAge for MS risk. **(C)** Forest figure of plasminogen activator inhibitor-1 for Alzheimer’s disease risk. **(D)** SNP effect on exposure of GrimAge on Parkinson’s disease. **(E)** SNP effect on exposure of HannumAge on MS. **(F)** SNP effect on exposure of plasminogen activator inhibitor-1 on AD.

Using an IVW approach, we observed a significant decrease in the risk of PD with GrimAge (OR = 0.8862, 95% CI 0.7914–0.9924, *p* = 0.03638) (**Figure 3A**). Consistently, negative associations between GrimAge and PD risk were also found using other methods such as maximum likelihood, weighted median, simple mode, and weighted mode (**Figure 3D**, **Table 1**, **Supplementary Table 1**).

**Table 1:** Causal effect of epigenetic age on neurodegenerative diseases.

Exposure	Outcome	Method	nSNP	*p*-value	OR(95%CI)
DNA methylation GrimAge acceleration	Parkinson’s disease	Maximum likelihood	4	0.016	0.883(0.799-0.977)
		MR Egger	4	0.355	2.690(0.530-13.661)
		Weighted median	4	0.077	0.897(0.794-1.012)
		Inverse variance weighted	4	0.036	0.886(0.791-0.992)
		Simple mode	4	0.226	0.857(0.702-1.046)
		Weighted mode	4	0.365	0.913(0.773-1.079)
DNA methylation HannumAge acceleration	Multiple Sclerosis	Maximum likelihood	5	0.033	1.072(1.006-1.142)
		MR Egger	5	0.526	1.136(0.801-1.611)
		Weighted median	5	0.011	1.111(1.024-1.024)
		Inverse variance weighted	5	0.033	1.071(1.006-1.140)
		Simple mode	5	0.121	1.135(1.000-1.289)
		Weighted mode	5	0.139	1.132(0.992-1.291)
DNA methylation-estimated plasminogen activator inhibitor-1 levels	Alzheimer’s disease	Maximum likelihood	4	0.006	1.000(1.000-1.000)
		MR Egger	4	0.751	1.000(1.000-1.000)
		Weighted median	4	0.145	1.000(1.000-1.000)
		Inverse variance weighted	4	0.044	1.000(1.000-1.000)
		Simple mode	4	0.490	1.000(1.000-1.000)
		Weighted mode	4	0.285	1.000(1.000-1.000)

SNP, single-nucleotide polymorphisms.

Additionally, we identified that HannumAge was linked to an increased risk of MS (OR = 1.0707, 95% CI 1.0056–1.1401, *p* = 0.03295) (**Figure 3B**). Similarly, the maximum likelihood, MR-Egger, weighted median, simple mode, and weighted mode methods also indicated positive associations between HannumAge and MS (**Figure 3E**, **Table 1**).

Furthermore, we also found that estimated plasminogen activator inhibitor-1(PAI-1) levels demonstrated an increased risk for AD (OR = 1.0001, 95% CI 1.0000–1.0002, *p* = 0.04425) (**Figures 3C, 3F**). Beyond that, we did not observe any causal associations between epigenetic age and neurodegenerative diseases.

### 3.3 Causal effect of neurodegenerative diseases on epigenetic age

**Figure 4:**
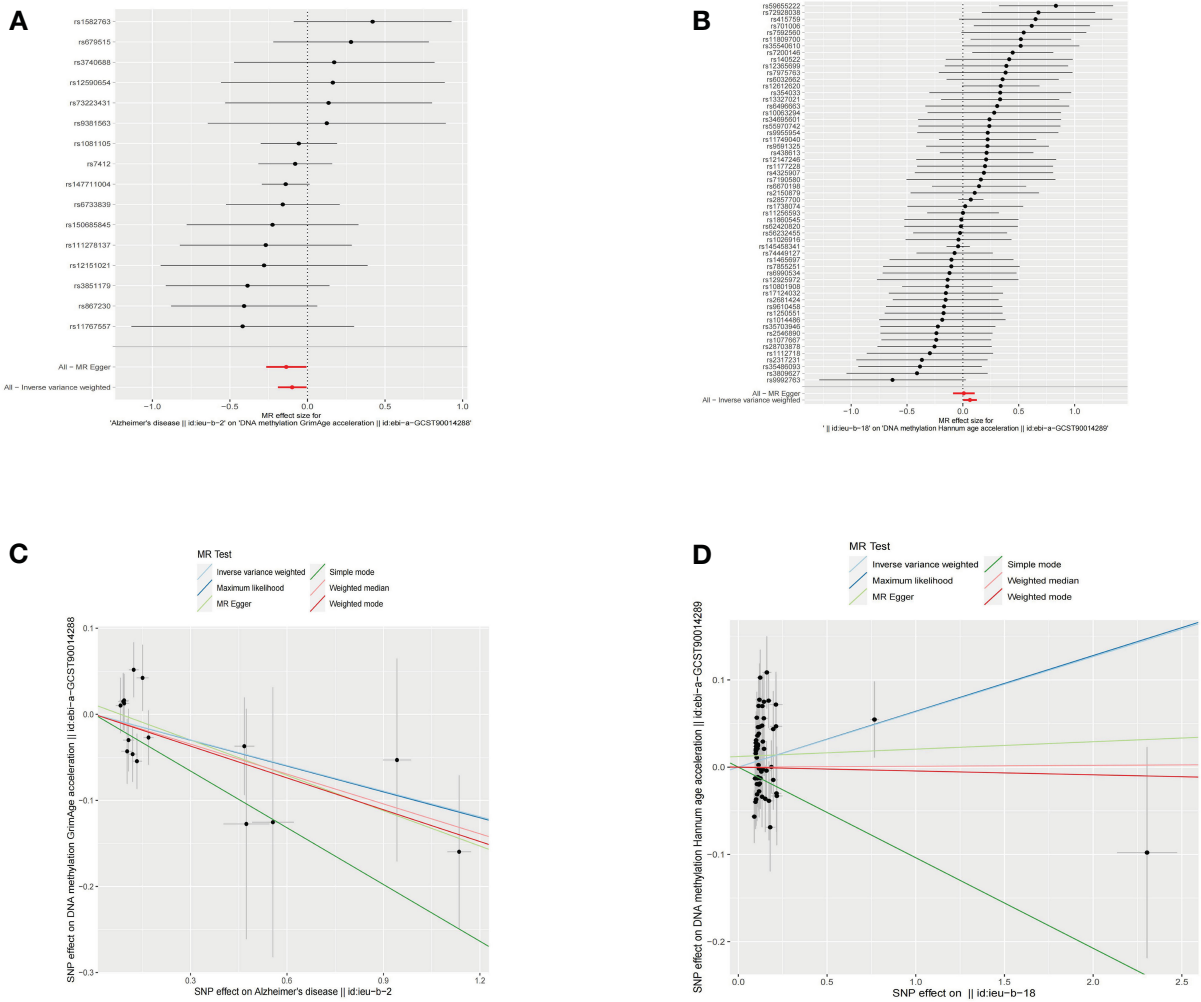
Causal effect of neurodegenerative diseases on epigenetic age. **(A)** Forest figure of AD for GrimAge. **(B)** Forest figure of MS for HannumAge. **(C)** SNP effect on exposure of AD on GrimAge. **(D)** SNP effect on exposure of MS on HannumAge.

Using an IVW approach, genetically determined AD was found to have causal effect on GrimAge (OR = 0.9057, 95% CI 0.8265–0.9925, *p* = 0.03386) (**Figures 4A, C**), while genetically determined MS has a causal effect on HannumAge (OR = 1.0653, 95% CI 1.0031–1.1314, *p* = 0.03932) (**Figures 4B, D**, **Table 2**, **Supplementary Table 1**).

**Table 2:** Causal effect of neurodegenerative diseases on epigenetic age.

Exposure	Outcome	Method	nSNP	*p*-value	OR 95%CI
Alzheimer’s disease	DNA methylation GrimAge acceleration	Maximum likelihood	16	0.033	0.905(0.825-0.992)
		MR Egger	16	0.056	0.872(0.767-0.992)
		Weighted median	16	0.063	0.891(0.788-1.006)
		Inverse variance weighted	16	0.034	0.906(0.827-0.993)
		Simple mode	16	0.046	0.803(0.658-0.979)
		Weighted mode	16	0.068	0.884(0.782-1.000)
Multiple Sclerosis	DNA methylation HannumAge acceleration	Maximum likelihood	57	0.015	1.066(1.012-1.123)
		MR Egger	57	0.863	1.009(0.917-1.110)
		Weighted median	57	0.98	1.001(0.922-1.087)
		Inverse variance weighted	57	0.039	1.065(1.003-1.131)
		Simple mode	57	0.335	0.901(0.731-1.111)
		Weighted mode	57	0.914	0.996(0.920-1.078)

SNP, single-nucleotide polymorphisms.

### 3.4 Sensitivity analyses

**Figure 5:**
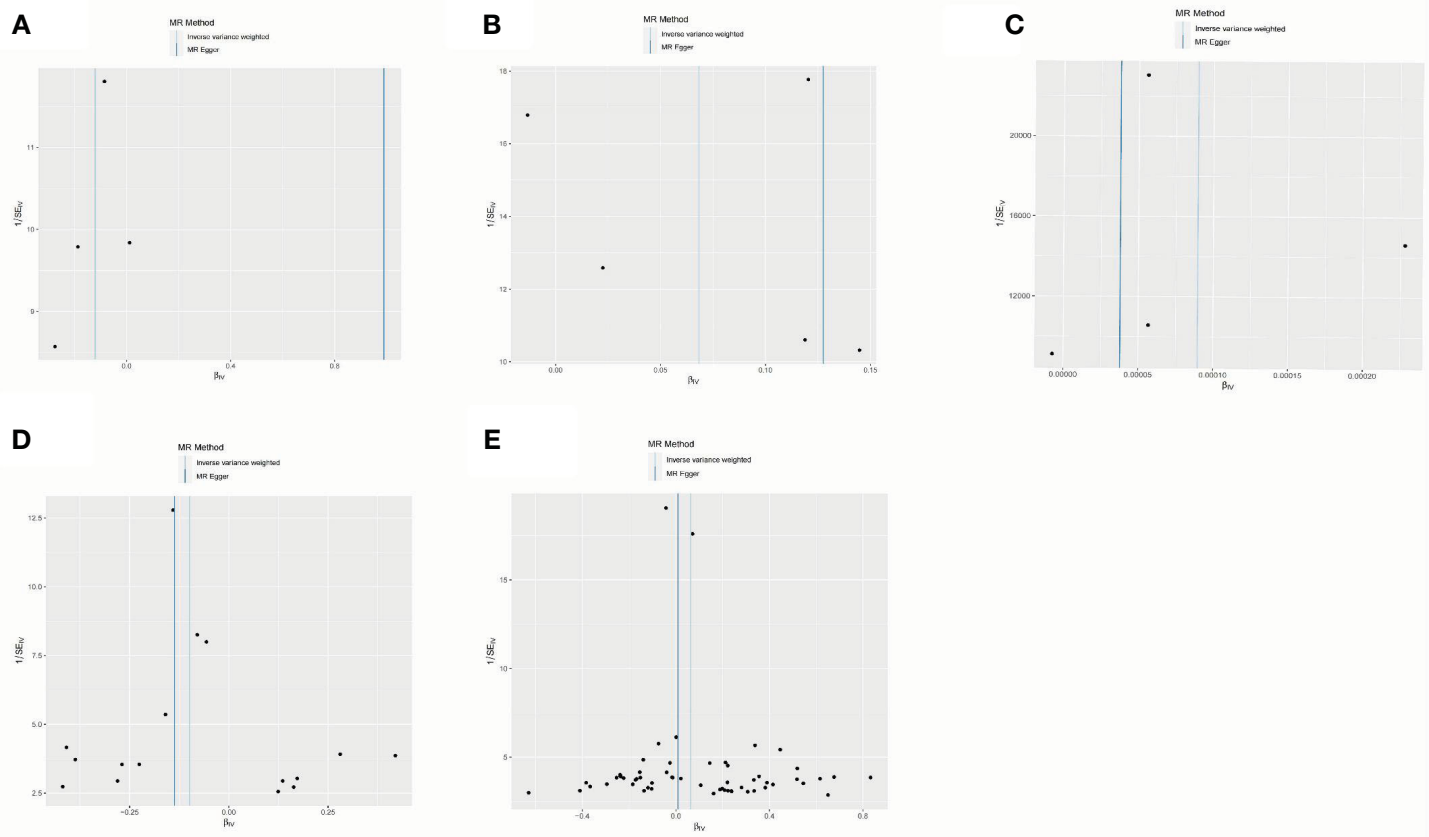
MR-Egger regression between epigenetic age and neurodegenerative diseases. **(A)** MR-Egger regression about risk of PD with GrimAge. **(B)** MR-Egger regression about risk of MS with HannumAge. **(C)** MR-Egger regression about risk of AD with plasminogen activator inhibitor-1. **(D)** MR-Egger regression about causal effect of AD on GrimAge. **(E)** MR-Egger regression about causal effect of MS on HannumAge.

**Figure 6:**
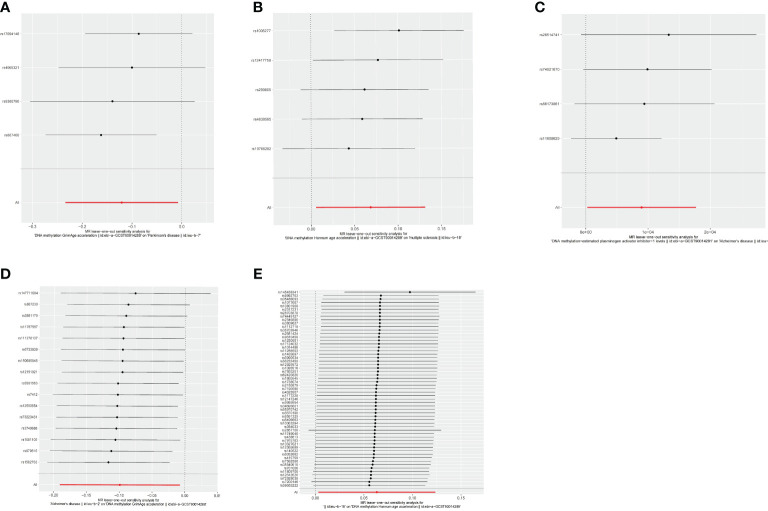
Leave-one-out analysis between epigenetic age and neurodegenerative diseases. **(A)** Leave-one-out analysis about risk of PD with GrimAge. **(B)** Leave-one-out analysis about risk of MS with HannumAge. **(C)** Leave-one-out analysis about risk of AD with plasminogen activator inhibitor-1. **(D)** Leave-one-out analysis about causal effect of AD on GrimAge. **(E)** Leave-one-out analysis about causal effect of MS on HannumAge.

The estimates of causal effects obtained from multiple analytical methods, including maximum likelihood, MR-Egger regression, weighted median method, simple mode, and weighted model methods, consistently yielded similar results in terms of both magnitude and direction. This consistent pattern enhances the reliability and confidence in our findings. Our analysis did not uncover substantial evidence of horizontal pleiotropy, suggesting that the instrumental variables utilized in the study were not influenced by factors other than the specific exposure of interest. This was supported by p-values greater than 0.05 when applying the MR-Egger regression intercept approach. Furthermore, the assessment of heterogeneity using Cochrane’s Q statistics did not reveal statistically significant differences among the estimates (*p* > 0.05). This indicates that the genetic variants employed as instruments for epigenetic age did not have significant differential effects on the outcome (**Figures 5A–E**, **Table 3**). Additionally, the leave-one-out analysis, where individual variants were systematically excluded, demonstrated the stability of the effect estimates and minimal influence from any single variant (**Figures 6A–E**). These findings further reinforce the robustness of our results. Taken together, these findings provide consistent and reliable evidence of a causal relationship between epigenetic age and neurodegenerative diseases, while also suggesting the absence of significant confounding factors or outliers affecting the observed effects (**Supplementary Table 2**).

**Table 3:** Sensitivity analyses of causal association between epigenetic aging and neurodegenerative diseases.

Exposure	Outcome	Heterogeneity				Horizontal pleiotropy		
		method	Q	Q_df	Q_pval	Intercept	SE	*p*-value
DNA methylation GrimAge acceleration	Parkinson’s disease	MR Egger	2.132	2	0.344	-0.21	0.15	0.312
		Inverse variance weighted	4.051	3	0.256			
DNA methylation HannumAge acceleration	Multiple Sclerosis	MR Egger	3.83	3	0.28	-0.017	0.049	0.757
		Inverse variance weighted	3.977	4	0.409			
DNA methylation-estimated plasminogen activator inhibitor-1 levels	Alzheimer’s disease	MR Egger	4.815	2	0.09	0.045	0.078	0.626
		Inverse variance weighted	5.599	3	0.133			

## 4 Discussion

Our study is the first to elucidate underlying association between aging and neurodegenerative diseases from an epigenetic aging perspective by bidirectional MR. Although there have been many studies exploring relationship of epigenetic aging on neurodegenerative disease, few studies focused on causal effect between them. The causal relationship between epigenetics and neurodegenerative diseases may contribute to identifying targets for treatment and prevention of neurodegenerative diseases (3). Our study selected PD, AD, and MS as representative neurodegenerative diseases and conducted an MR study in combination with the latest published epigenetic aging biomarkers. Our study involves a PD source (33,674 cases, and 449,056 controls),AD data (21,982 cases and 41,944 controls),and an MS source (47,429 cases and 68,374 controls). We found evidence to support the claim that there is causality between epigenetic aging and neurodegenerative diseases. Epigenetic aging may act as an one of the triggers to determine the incidence and prevalence of neurodegenerative diseases.

It is commonly known that aging is a major hazard for neurodegenerative diseases, a deteriorating brain is an arbiter of neurodegenerative diseases (3). And significant progress has been made in gene discovery and gene therapy for neurodegenerative diseases. However, neurodegenerative diseases may not be simply viewed as results of accelerated aging or gene disorder (3). There is growing evidence that epigenetic age and neurodegenerative diseases are related. Several studies have shown that epigenetic age is accelerated in brains and blood of patients with neurodegenerative diseases, compared to healthy controls (22). One possible mechanism for this relationship is that epigenetic aging may affect the expression of genes that are involved in neuronal function, survival, and repair. For example, epigenetic aging may alter the expression of genes that regulate inflammation (23), oxidative stress (24), synaptic plasticity (25), and neurogenesis (26). Additionally, neurodegenerative diseases may also cause acceleration of epigenetic aging. For example, neurodegenerative diseases may induce DNA damage (27), oxidative stress (28), inflammation (29), and metabolic dysfunction (30), which can affect DNA methylation patterns. However, various calculation methods of epigenetic aging and different kinds of neurodegenerative diseases have both homogeneity and heterogeneity.

As for PD, we observed a negative association between GrimAge and risk of PD. GrimAge is the second of epigenetic clocks, which is calculated based on various biomarkers, such as DNA methylation, telomere length, and blood biochemistry (31). GrimAge is considered to be a more accurate predictor of mortality and health outcomes than chronological age (31). One study has confirmed that GrimAge is associated with grip strength, walking speed, polypharmacy, and all-cause mortality (31). Even though PD is classified as a type of neurodegenerative disorder, the mortality rate is increasing annually (32). The cause of death from PD has long been debatable and elusive, and there are significant individual variances in mortality rates among patients. According to one recent research, late onset age, dementia, cardiac abnormalities and autonomic dysfunction have been viewed as triggers leading to an increase in mortality rate (33). Our results showed a negative associations between GrimAge and PD risk. In neurodegenerative diseases, this may mean that GrimAge has specific predictive ability for mortality of PD. Predicting the mortality rate of PD can contribute to personalized treatment and prevention strategies for PD.

MS is a chronic disease causing demyelinating, neurodegenerative lesions to the central nervous system (34). MS is believed to have an autoimmune disorder in pathology and progressive mechanism, with polygenic and environmental susceptibility factors (35). However, pathology and progressive mechanism of MS cannot be solely attributed to genetic or environmental factors.

From view of the progressive mechanism, the clinical stage of MS is better explained as a continuum that may vary according to the amount of individuals (36). MS has always been categorized by distinct clinical stage—relapsing/remitting(RRMS), secondary/progressive(SPMS), and primary/progressive(PPMS) (36). Personalized progression continuum may be related to a transition from predominantly localized injury to widespread inflammation and neurodegeneration (36, 37).According to recent research, aging mainly affects the progression of MS in conjunction with the immune system and microbiota (38, 39). Deregulation of the immune system caused by aging directly hinders the repair pathways in progressive MS. Like concentrations of the chemokine eotaxin (CCL11) in blood and CSF, which are associated with clinical disability and radiological lesion burden in patients with MS (40). The microbiota and immune system reciprocally affect each another. Aging amplifies the process in which immunoregulatory microbial products diffuse into circulation from the gut through increased intestinal epithelial permeability (40). Similarly, aging is associated with a reduction in fecal concentrations of short chain fatty acids, which might exacerbate the immune system and MS progression. The process of aging is linked to a decrease in levels of short chain fatty acids in feces, perhaps leading to changes in the immune system and worsening the progress of MS (41). Comparing biological changes with the risk of aging-related diseases and mortality by measuring epigenetic clocks has been viewed as promising method. Compared with healthy control participants, one study showed that epigenetics can unveil higher age acceleration in patients with MS (42). But the association of this metric with progression of MS was not clear.

Of interest, we identified that HannumAge was linked to an increased risk of MS. This may support the idea that HannumAge is a specific risk factor for MS in different types of multiple sclerosis. Hannum et al. analyzed DNA methylation patterns from two cohorts’ whole blood data to discover 71 CpG sites that might be used to calculate age, which is called HannumAge (43). HannumAge estimator, the first epigenetic clock, is considered to track aspects of immunosenescence. HannumAge has been described as a biomarker of immune system aging (44). One study found that Hannum is positively correlated with estimated abundance of exhausted, plasmablast cells, and is negatively correlated with naive CD8+ T cell types (45). Since pathogenic B and T cells are prone to enter the central nervous system, interacting B and T cells drive the pathogenesis of MS (46). Genetic burden and environmental factors may contribute to the CNS-infiltrating (46, 47). HannumAge has been found to be associated with sensitivity to variations in environment and lifestyle (48).

Current evidence indicates that MS pathogenesis should integrate underlying risk related to genetic susceptibility with epigenetic age (49). Our result may indicate that HannumAge may affect pathogenesis and progression of MS by regulation of immunity and inflammation, which is consistent with the previous point. Epigenetics emerges as a crucial intermediary factor that bridges the gap between genes and the environment factors. Therefore, Hannum’s age may be a potential research direction for preventing progression and treatment of MS in response to epigenetic aging and environmental factors.

Significantly, we also found that MS can in turn affect acceleration of the Hannum clock. There is currently a lack of research on acceleration of Hannum’s age. According to the present study, possible factors that can accelerate Hannum epigenetic age mainly include smoking, obesity and inflammation. Inflammatory markers in blood were always associated with higher Hannum epigenetic age (50). As mentioned before, progression of MS is gradual and can be divided into three stages(RRMS,SPMS,PPMS). Inflammation plays an important role in progression of MS to varying degrees (51). Therefore, results shows there is mutual causality in a continuous process of onset and progression of MS. Hannum clock may lead to onset of MS through immune system disorders. Afterwards, diffusion of inflammatory reactions leads to an acceleration in the Hannum clock as MS progresses.

According to numerous research works, several age-related diseases are linked to a dysfunctional fibrinolytic system (52). Plasminogen activator inhibitor-1 (PAI-1) is the primary physiological inhibitor of both tissue type and urokinase type plasminogen activators. Therefore, it is the main regulator of plasminogen activation system activators (53). According to recent research, PAI-1 has both direct and indirect effects in the development of AD (54). Directly, accumulation of amyloid beta (Aβ) peptide was considered as the main pathogenesis of AD. Elevated expression of PAI-1 has been shown to interfere with plasmin-mediated clearance and degradation of Aβ, thereby contributing to neurotoxic plaques in AD (55–57). Additionally, inhibition of PAI-1 activity can reduce accumulation of Aβ and affect synaptic function in the brain, which promotes memory in AD model mice (58). A study has shown that Aβ might impair BDNF proteolytic processing through regulation of PAI‐1, which unveils potential mechanisms.

Indirectly, plasminogen activation system activators exhibit multiple functions that are potential risk factors for AD onset and progression, like diabetes, cardiovascular health, and chronic inflammation (52). Additionally, some studies further showed that increased PAI-1 expression may drive astrocyte senescence and that senescent astrocytes can promote neuron apoptosis by secreting PAI-1. In conclusion, there are increasing studies exploring the relationship between PAI-1 and AD (58–60). The present mendelian randomization study, *in vitro* and *in vivo* experiments have confirmed that PAI-1 is a biomarker of cellular aging (54, 61). PAI-1 may have a potential effect between AD and aging.

Our study is the first to confirm casual effect between AD and PAI-1 by MR, and we only found that estimated PAI-1 levels demonstrated an increased risk of AD. By contrast, we found no effect of AD on PAI-1. Therefore, our study suggests that PAI-1 may be a pathogenic factor of AD, instead of a result of AD onset. According to the present study, PAI-1 expression increases in plasma and brain of both AD patients and AD model mice (54, 62–64).

There are some kinds of molecules that were proven to be efficient PAI-1 inhibitors both *in vitro* and *in vivo (* 57, 58, 65–67). However, no PAI-1 inhibitor is presently licensed for use in humans. Our research is consistent with current research results and is the first to further confirm from a genetic perspective.

As significant DNAm-based surrogate markers, PAI-1 were causally influenced by lifestyle factors and had a causal effect on aging and disease outcomes (68–70). One study has shown that lifestyle intervention can reverse insulin-induced vascular dysfunction in parallel with decreased PAI-1 level (68). Another study has shown that exercise ameliorates PAI-1 mediated cardiovascular inflammation in renal insufficiency (71). However, there is currently no research focusing on how lifestyle can influence AD by reversing PAI-1. Our research findings indicate that it may be advisable to explore interventions aimed at reversing PAI-1 expression in order to achieve therapeutic or preventative effects on AD.

Although we found no effect of AD on PAI-1, our results show that AD had a causal effect of on GrimAge. Unlike other epigenetic ages, GrimAge is calculated from a formula that uses the methylation levels of 103 CpG sites, which are regions of DNA where a cytosine nucleotide is followed by a guanine nucleotide (31). GrimAge is also calculated based on various biomarkers, such as DNA methylation (31), telomere length(TL) (72, 73), and blood biochemistry (73–75). Among various triggers that affect GrimAge, DNA methylation measures of aging have offered promise, but their relation to AD has been equivocal. One cohort study showed that candidate CpG sites and regions in peripheral blood were identified as associated with the rate of cognitive decline (75). However, based on existing studies, one systematic review showed that DNA methylation is not associated with risk of dementia (17).

These equivocal findings, together with our results, imply that GrimAge may not be the factor causing AD. More likely, onset and development of AD may cause alterations in DNA methylation, resulting in an acceleration of GrimAge.

TL is a potential indicator of biological aging and age-related consequences (76, 77). Shorter telomeres are associated with higher overall risk of developing AD (78). However, previous literature showed inconsistent findings regarding TL effect on AD. Hägg et al. reported an association between longer TL and cognitive performance (79). Fani et al. found a kind of U-shaped association between TL and risk of AD (80), which means that both shorter TL and longer TL were associated with risk of AD. Blanca et al. did not find a statistically significant association between genetically longer TL and cognitive function (81). Differences between these results may be due to characteristics of cross-sectional and cohort studies. As one of the calculation tools for GrimAge, TL may be related to acceleration of GrimAge caused by AD onset. However, our study did not make specific analysis of causal relationship between TL and AD, further studies are needed to explore whether onset and progression of AD will lead to TL changes.

Besides TL and DNA methylation, GrimAge incorporates plasma protein levels into a composite measure of biological age. Several plasma proteins have association with GrimAge: beta-2-microglobulin (β_2_M) (82), adrenomedullin (83), cystatin C(Cys C) (84), PAI-1 (54). These proteins not only serve as the basis for calculating biological aging in GrimAge, but are also highly correlated with pathological mechanisms of AD. β_2_M (85), and Cys C (86) has been verified to be related to deposition of Aβ, the most discussed pathogenesis of AD.

β2M has been viewed as a coaggregation factor with Aβ (85). Increased soluble β2M has been detected in plasma with AD patients (73). Additionally, one study showed that targeting peripheral β_2_M can effectively decrease deposition of β-amyloid in brain of AD model mice and improve learning and memory abilities (87).

Cystatins are a family of cysteine protease inhibitors, which play a significant role in regulating abnormal accumulation of Aβ in AD (88). Wang et al. have shown that plasma Cys C levels were higher in patients with AD than in healthy subjects (84). And there is correlations between plasma Cys C levels and severity scores in AD patients (84).

Adrenomedullin has also been shown to have diagnostic value for AD (83). In addition, Buerger et al. shows that plasma concentrations of adrenomedullin have predictive value in progression from MCI to clinical AD (89), which may indicate adrenomedullin is a biomarker of AD progression. As we have shown, AD has causal effect on GrimAge, and it is suggested that calculating GrimAge with adrenomedullin may help predict progression of AD.

Changes of GrimAge related proteins in plasma of AD patients may be one of the reasons for GrimAge increase caused by progression of AD. Therefore, calculating GrimAge as a result in future studies may be useful in predicting prognosis and progression of AD.

Additionally, a clinical study found that the GrimAge predictor of human morbidity and mortality showed a 2-year decrease in epigenetic vs. chronological age after intervention (90). These preliminary results confirmed that epigenetic GrimAge aging is reversible, which sheds light on potential epigenetic therapy for AD.

In conclusion, our study is the first to elucidate underlying association between aging and neurodegenerative diseases from an epigenetic aging perspective by using bidirectional MR. We found negative associations between GrimAge and PD, PAI-1 and AD. We also found a casual effect between HannumAge and MS, as well as GrimAge and AD. This suggests that epigenetic age and neurodegenerative diseases may have a bidirectional relationship, where each one influences the other. Therefore, understanding this relationship may help to identify biomarkers for early diagnosis, prognosis, and treatment. It may also reveal novel targets for interventions that can slow down or reverse epigenetic aging and prevent or delay neurodegeneration.

Our study is not free of limitations. From the perspective of clinical significance, neurodegenerative diseases are multifaceted, involving genetic, epigenetic, and environmental factors. Epigenetic clocks, although offering valuable insights, provide but just one piece of the puzzle in understanding the complex mechanisms. Further research is needed to determine whether epigenetic clocks lead to the occurrence of neurodegenerative diseases through interactions with other causes. Methodologically, there are many determining factors for each generation epigenetic clock. Although this study has confirmed correlation between some epigenetic clocks and neurodegenerative diseases in a broad sense, it has not targeted specific determining factors, which also highlights a need for further studies. Types of neurodegenerative diseases are not limited to PD, AD, and MS. Our study did not include all types of neurodegenerative diseases. Further research on the correlation between other types of neurodegenerative diseases and epigenetic aging are conducive to find promising candidate for diagnosis and prognosis.

Cancer and aging are accompanied by changes to epigenetic clocks, including progressive loss of DNA methylation over gene-poor genomic regions (91). Much evidence demonstrated that modifications in genetic and epigenetic characteristics may drive tumor metastasis (92). The presence of intricate inter- and intratumor heterogeneity is a characteristic observed in several cancers, frequently leading to limited efficacy of treatment interventions (93). Epigenetic changes, due to their dynamic and reversible character, have great potential as a target for innovative anticancer treatments. In future research, the study of cancer incidence, metastasis, and treatment resistance through epigenetic perspectives may provide more profound implications. Besides, epigenetics is a crucial factor in the pathogenesis of several metabolic disorders, such as diabetes, obesity, and osteoporosis (94). Enhanced comprehension of epigenetic regulatory systems in metabolic illnesses facilitates a comprehensive understanding of these conditions, consequently enabling the development of innovative therapeutic approaches.

The research subjects included in this study were mostly from the United States and Europe. In subsequent research, it will be necessary to promptly follow up on data updates from multiple races for analysis, in order to obtain more representative conclusions.

Unlike genetics, epigenetic changes are mostly reversible. Therefore, epigenetics has a great potential for clinical application. We provided primary evidence that epigenetic aging might be a potential diagnostic or patient-tailored therapeutic approach for neurodegenerative diseases through MR research, but further extensive research is still required.

## Data availability statement

The original contributions presented in the study are included in the article/**Supplementary Material**. Further inquiries can be directed to the corresponding author.

## Author contributions

JF: Conceptualization, Methodology, Software, Writing – original draft. QL: Conceptualization, Writing – review & editing. XL: Conceptualization, Data curation, Methodology, Software, Writing – original draft. MG: Conceptualization, Investigation, Software, Writing – review & editing. IL: Investigation, Software, Writing – review & editing. YT: Software, Writing – review & editing. XX: Data curation, Methodology, Writing – review & editing. LS: Data curation, Writing – review & editing. LD: Software, Writing – review & editing. YZ: Methodology, Writing – review & editing. ML: Formal analysis, Project administration, Writing – review & editing. LZ: Funding acquisition, Validation, Writing – review & editing.

## Supplementary material

Supplementary Table 1 Causal effect between epigenetic age and neurodegenerative diseases.

Supplementary Table 2 Sensitive analysis_Causal effect between epigenetic age and neurodegenerative diseases.

Supplementary Table 3 Instrumental variables_Causal effect of epigenetic age on neurodegenerative diseases.
